# Phytomicrobiome Research for Disease and Pathogen Management

**DOI:** 10.3390/plants14060947

**Published:** 2025-03-18

**Authors:** Adeline Picot, Wen Chen

**Affiliations:** 1 University of Brest, INRAE, Laboratoire Universitaire de Biodiversité et Écologie Microbienne, F-29280 Plouzané, France; adeline.picot@univ-brest.fr; 2 Ottawa Research and Development Centre, Science and Technology Branch, Agriculture and Agri-Food Canada, Ottawa, ON K1A 0C6, Canada; 3 Department of Biology, University of Ottawa, Ottawa, ON K1N 6N5, Canada

Microorganisms associated with soil and plants, also known as the phytomicrobiome, include beneficial members that provide critical ecosystem services and pathogens that threaten food safety and security. Despite the well-established roles of the phytomicrobiome in disease onset and development, research has primarily focused on behaviours of specific microorganisms (pathogens or biocontrol agents) rather than the microbiome as a whole. The advent of high-throughput omics tools, exemplified by amplicon or shotgun-based metagenomics, metatranscriptomics, metaproteomics, and metabolomics, has empowered our ability to explore microbial diversity and functionality. These technologies enable the identification of bioindicators (microorganisms, genes, and/or molecules) associated with ecosystem goods and services, reveal synergism and antagonism interactions across kingdoms, and assess the impact of complex environmental factors induced by climate change and anthropogenic land uses. Integrating this knowledge with wet-lab and field validations presents opportunities for developing innovative biocontrol strategies and improving pathogen management to support plant and soil health. Additionally, this research area addresses key challenges in advancing sustainable farming practices.

This Special Issue comprises four review articles and six research papers, showcasing the latest and most significant insights in the soil and phytomicrobiome in relation to plant disease management. This collection features an Editor’s Choice paper—a review by the guest editors examining how agricultural practices influence soil health and pathogen suppression through phytomicrobiome modulation under changing climate scenarios [1]. The authors discussed both the conceptual and computational challenges of characterizing and interpreting phytomicrobiomes, emphasizing the need for rigorous assessment of microbial inoculants to ensure their efficacy, safety, long-term ecological impacts, and resilience to climate-induced disruptions. Another review by Zhao et al. [2] explored artificial intelligence (AI)-based tools for analyzing microbiome diversity and functionality, phenotyping and forecasting plant diseases, and integrating multidisciplinary data. The authors highlighted the transformative potential of AI, particularly machine learning and deep learning, in addressing challenges such as data complexity and predictive modelling, ultimately advancing sustainable agriculture through enhanced plant health and disease management strategies. Two additional review articles focused on the biological control of agriculturally important diseases. Orozco-Mosqueda et al. [3] highlighted the potential of plant growth-promoting bacteria (PGPB) as eco-friendly biocontrol agents for mitigating grey mould (*Botrytis cinerea*), while Saleem et al. [4] explored the diverse applications of *Serendipita indica* in enhancing plant adaptability, health, and development, positioning it as a promising tool for sustainable farming.

This Special Issue also includes six original research articles covering diverse topics. Plessis et al. [5] introduces ASVmaker, a computational tool designed to compile reference databases from multiple public repositories, enhancing taxonomic classification accuracy of short high-throughput sequencing reads—an urgent need in phytopathogen detection using metabarcoding. Belair et al. [6], on the other hand, compared the performance of different primer sets and bioinformatics pipelines in recovering walnut dieback fungal pathobiome. Two studies focused on how agricultural practices, such as tillage and crop rotations [7] as well as fungicide applications [8], significantly impacted soil microbiomes and pathogen dynamics. These studies shed light on both the beneficial and negative consequences of these farming strategies in soil health and disease management. By examine changes in root endophytic and rhizosphere microbiomes in response to two pathotypes of *Plasmodiophora brassicae* (the causal agent of clubroot in canola), Cordero-Elvia et al. [9] searched for potential biological control agents for this soilborne disease. Last but not the least, Hoffman et al. [10] turned their focus to phyllosphere fungal communities, an understudied subject, and identified taxonomic biomarkers across regional wheat production areas in northeastern Germany that may guide the development of adaptive agricultural strategies.

In conclusion, this Special Issue provides a comprehensive overview and targeted studies that highlight advancements in soil and phytomicrobiome research, offering readers valuable insights into the critical role of the microbial communities in agricultural production systems. The findings underscore the importance of further exploring of phytomicrobiome members as potential solutions for combating phytopathogens and improving soil and plant health. These studies also demonstrated the impact of various cultivation practices on the phytomicrobiome and its potential to support sustainable crop production. While an array of omics technologies has enhanced our understanding of phytomicrobiome composition and function, further research, ranging from computational and wet-lab studies to field investigation, is needed to unravel the complex microbial interactions within these communities and their roles in disease progression. We recognize the critical importance of developing and advancing AI-based tools capable of integrating multidisciplinary data to revolutionize phytopathogen management. These tools hold immense potential for translating fundamental mechanistic and ecological knowledge into highly accurate, actionable predictions of soil and phytomicrobiome functioning. By enabling real-time monitoring of soil health and crop quality through phytomicrobiome research, it is possible to develop enhanced disease forecasting, optimized biocontrol strategies, and improved sustainable farming practices, ultimately paving the way for the transformation of agriculture through digital technologies. The editors emphasize that although this Special Issue focuses on pathogen management, soil and the phytomicrobiome also play a crucial role in alleviating abiotic stresses such as extreme temperature, drought, and hypersalinity, which are expected to intensify due to changing climate. Therefore, evaluating the protective capacities of soil and phytomicrobiome members against both biotic and abiotic stresses is essential for developing resilient agricultural systems and ensuring long-term crop sustainability in the face of climate change and agricultural intensification.
